# Role of STAT3 pathway in genitourinary tumors

**DOI:** 10.4155/fso.15.13

**Published:** 2015-11-01

**Authors:** Matteo Santoni, Alessandro Conti, Francesco Piva, Francesco Massari, Chiara Ciccarese, Luciano Burattini, Liang Cheng, Antonio Lopez-Beltran, Marina Scarpelli, Daniele Santini, Giampaolo Tortora, Stefano Cascinu, Rodolfo Montironi

**Affiliations:** 1 Medical Oncology, Università Politecnica delle Marche, Azienda Ospedaliero-Universitaria Ospedali Riuniti Umberto I, GM Lancisi, G Salesi, Ancona, Italy; 2 Department of Specialistic Clinical & Odontostomatological Sciences, Section of Urology, Polytechnic University of Marche, Ancona 60126, Italy; 3 Department of Clinical Sciences, Polytechnic University of the Marche Region, Piazza Roma 22, 60121 Ancona, Italy; 4 Medical Oncology, Azienda Ospedaliera Universitaria Integrata, University of Verona, Verona, Italy; 5 Department of Pathology & Laboratory Medicine, Indiana University School of Medicine, 635 Barnhill Drive, Indianapolis, IN 46202, USA; 6 Department of Surgery, Cordoba University Medical School, Cordoba, Spain; 7 Section of Pathological Anatomy, Polytechnic University of the Marche Region, School of Medicine, AOU Ospedali Riuniti, Ancona, Italy; 8 Medical Oncology Department, University Campus Bio-Medico, Rome, Italy

**Keywords:** drug resistance, genitourinary tumors, metastasis, signal transducer and activator of transcription 3, tumor microenvironment

## Abstract

The *STAT3* is often dysregulated in genitourinary tumors. In prostate cancer, *STAT3* activation correlates with Gleason score and pathological stage and modulates cancer stem cells and epithelial–mesenchymal transition. In addition, *STAT3* promotes the progression from carcinoma *in situ* to invasive bladder cancer and modulates renal cell carcinoma angiogenesis by increasing the expression of HIF1α and VEGF. *STAT3* is also involved in the response to tyrosine kinase inhibitors sunitinib and axitinib, in patients with metastatic renal cell carcinoma, and to second-generation androgen receptor inhibitor enzalutamide in patients with advanced prostate cancer. In this review, we describe the role of *STAT3* in genitourinary tumors, thus describing its potential for future therapeutic strategies.

## Abstract (lay abstract)

Regulation of the protein *STAT3* is often dysfunctional in genitourinary tumors, including prostate, bladder and kidney cancer. *STAT3* is involved in the generation of these tumors and has an effect in their response to treatments. In this review, we describe its role in genitourinary tumors and discuss its potential for use in future therapies.

**Figure 1.**
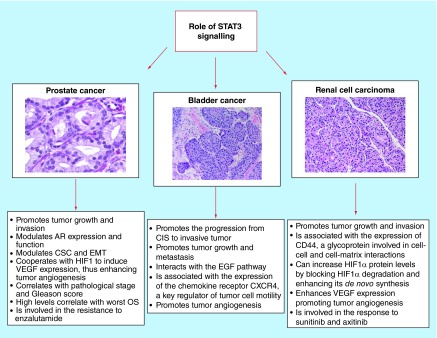
**Role of STAT3 in genitourinary tumors.** AR: Androgen receptor; CIS: Carcinoma in *situ*; CSC: Cancer stem cell; EMT: Epithelial–mesenchymal transition; OS: Overall survival.

The STAT proteins are involved in mediating cellular responses to cytokines [1]. STAT family includes seven members (STAT1, STAT2, STAT3, STAT4, STAT5A, STAT5B and STAT6). Among them, STAT3 plays a prominent role in tumor growth and invasion [2]. STAT3 was discovered as an acute phase response factor, due to its ability to increase the expression of liver proteins in response to stress [3]. STAT3 activation (pSTAT3) is determined by the phosphorylation of the tyrosine residue at position 705 by JAK [4]. Once activated, pSTAT3 forms dimers, translocates into the nucleus, and binds to STAT-specific DNA-response elements, called gamma-activated sites to promote the transcription of selected genes.

STAT3 presents three alternative splice isoforms: the isoform 1 or α (producing a 770 aminoacids protein), the isoform 2 (lacking aminoacid 701) and the isoform 3 or β (lacking AAs 723–770 and with a shift from TTCSNTI to FIDAVWK, corresponding to aminoacids 716–722). STAT3α is the more frequent isoform expressed in human carcinomas and is involved in tumor development and metastasis. Otherwise, the β isoform seems to inhibit the transcriptional activation due to α isoform, resulting in tumor growth inhibition [5,6].

The list of STAT3 activators includes IL-6, EGF, S1P, inflammatory OSM, Src family members and growth factor receptors that possess intrinsic tyrosine-kinase activity, such EGFRs, HGF receptor (also known as c-Met) and PDGFR [7–10].

Deregulation of STAT3 signaling has been reported in several solid tumors, including breast [11], head and neck [12], prostate, renal, bladder, pancreas, ovarian and brain cancers and melanoma [13–19]. This review summarizes recent findings on the role of STAT3 in tumor initiation, progression and angiogenesis, focusing on genitourinary tumors.

## Role of STAT3 signaling in cancer

STAT3 acts as transcriptional regulator of a variety of tumor-promoting genes. Persistent STAT3 activation may be due to the loss of suppressors of cytokine signaling and protein tyrosine phosphatases expression, as well as to autocrine or paracrine inflammatory stimulation in the tumor microenvironment.

STAT3 activation alone is sufficient to induce cell transformation, showing a strong oncogenic potential and promotes the maintenance of a procarcinogenic inflammatory microenvironment during cancer initiation and progression [20–22]. In addition, STAT3 inhibition has been shown to suppress tumor growth and enhance the sensitivity to drugs in a variety of solid tumors [23,24].

The list of STAT3 target genes includes *VEGF, Bcl2, c-myc, cyclin D1, Survivin* and *WASF3*, a member of the WASP/WASF family, which are involved in tumor development and progression [25–29]. *WASF3* regulates cell motility by modulating actin cytoskeleton dynamics [66] and promotes invasion through the activation of NFκB and ZEB1 [30]. Furthermore, STAT3 promotes carcinogenesis through the chaperone protein aging-associated gene 8 protein, which contributes to endoplasmic reticulum-associated degradation and promotes carcinogenesis both *in vitro* and *in vivo* [31].

The epithelial–mesenchymal transition (EMT) is a process by which epithelial cells transdifferentiate into motile mesenchymal cells. Inappropriate activation of embryonic EMT programs in cancer cells promotes cell plasticity and invasion [32]. Moreover, EMT is implicated in the acquisition of stem cell-like and chemoresistant phenotypes by tumor cells [33]. Interestingly, STAT3 promotes cancer invasion also by modulating EMT [34]. Indeed, STAT3 regulates the expression of transcriptional factors driving EMT and modulates cytoskeleton dynamics during the initiation of the EMT process [34].

Recent studies have revealed persistent STAT3 activation in myeloid and T cells at primary tumor sites contribute to tumor-related immunosuppression, angiogenesis, growth and metastasis [35–39]. In addition, STAT3 signaling plays a crucial role also in other types of stromal cells, such as fibroblasts and endothelial cells, in initiating premetastatic niche formation [40].

## Role of STAT3 signaling in prostate cancer

Prostate cancer (PCa) is one of the leading causes of death among men. In the last years, major advances have been made in understanding the genetic mechanisms underlying PCa. *STAT3* plays a crucial role in prostate carcinogenesis [41,42], as sustained by the evidence that *STAT3* knockdown is associated with inhibited tumor growth in preclinical models [43]. STAT3 signaling is involved in modulating PCa cell survival. Indeed, STAT3 is required for the activation of antiapoptotic proto-oncogenes, such as Bcl-2 and Bcl-3 [44–46] and for the modulation of androgen receptor (AR) expression and activity [47]. Furthermore, *in vitro* studies showed that STAT3 activation is higher in androgen-insensitive DU145 and PC3 cell lines compared with androgen-sensitive LNCaP cells [48], suggesting for a role of this pathway in the modulation of AR activity [48].

The role of STAT3 signaling in modulating prostate cancer stem cells (PCSCs), EMT and tumor angiogenesis has only recently been investigated (Figure 1). PCSCs have been found in both prostate [49] and PCa. Their presence is associated with high STAT3 activity, low AR expression, higher potential to metastasize and with poor patient outcome [50]. It has been shown that treatment with LLL12, a STAT3 inhibitor, abrogates the propagating of PCSCs *in vivo* [51]. Furthermore, STAT3 knockdown inhibits sphere formation derived from human PCa cells [43] The crucial role of STAT3 in PCSCs may be explained by its role in the IL-6 signaling, as sustained by the notion that soluble IL-6 receptor fusion protein can significantly reduce CSC number and xenograft tumor growth in *in vivo* PCa models [43].

STAT3 activators IL-6 and CCL2 chemokine have been shown to play a role in modulating EMT in PCa. The EGF is also involved in EMT programs. The activity of EGF is mediated by several pathways, including STAT3, hypoxia inducible factor (HIF)1α and TWIST1 [52]*.* TWIST1 is a highly conserved transcription factor that belongs to the basic helix–loop–helix family [53] and represents a key step during PCa development and metastasis due to its role in EMT [54,55]. Notably, TGF-β1 has been shown to upregulate TWIST1, as well as to promote STAT3 activation and HIF1α stabilization, thus contributing to PCa EMT and metastasization [55].

Interestingly, STAT3 is also implicated in promoting PCa angiogenesis [56]. Indeed, STAT3 is required for VEGF signaling [57]. The relationship between STAT3 and AR in PCa is crucial in modulating *VEGF* transcription, may be due to the presence of AR-binding sites in the promoter of *VEGF* gene [58]. Moreover, STAT3-induced HIF1α transcription, which cooperates with STAT3 to induce *VEGF* expression [59].

Activated STAT3 signaling is associated with the clinicopathologic characteristics of PCa, such as high pathological stage and Gleason score [60,61]. In addition, the expression levels of STAT3 activator IL-6 are increased in patients with metastatic PCa compared with patients with nonmalignant diseases [62–64].

STAT3 activation negatively correlated with overall survival (OS) in PCa patients from biochemical relapse [61] and in castration-resistant PCa patients [65]. Furthermore, activated STAT3 is associated with shorter recurrence-free survival in patients who undergo radical prostatectomy or hormonal therapy [61].

Interestingly, STAT3 is involved in the development of drug resistance in patients with PCa. Enzalutamide is a second-generation AR inhibitor. Enzalutamide has been showed to increase the OS of patients with metastatic PCa both in chemo-naive [66] and in patients pretreated with chemotherapy [67]. The identification of the mechanisms underlying primary and acquired resistance to this agent represents a major challenge for uro-oncologists. Antonarakis *et al*. reported that the presence of AR isoform encoded by splice variant 7 (AR-V7), which is constitutively activated but lacks of the ligand-binding domain targetable by abiraterone and enzalutamide, was associated with drug resistance in 62 patients treated with one of these two agents [68]. In addition, the downregulation of STAT3 seems to reverse the resistance to enzalutamide in PCa cells. Thus, the combination of STAT3 inhibitor AG490 and enzalutamide significantly inhibited tumor growth and induced cell apoptosis [69].

Furthermore, the inhibition of STAT3 signaling using small-molecule inhibitor Stattic has been shown to target both tumor-initiating cells (TICs) and differentiated cells. In this study, STAT3 inhibition caused S-phase accumulation at low-dose levels and massive apoptosis at a relatively high-dose level in PCa cells. STAT3 knockdown led to the disruption of the microvascular niche which TICs and non-TICs depend on. Thus, STAT3 inhibition is predicted to have greater efficacy for PCa treatment [70].

## Role of STAT3 signaling in bladder cancer

Bladder cancer (BC) is the fourth most frequent cancer in men in developed countries [71]. Two main distinct forms based on the infiltration of the muscularis propria have been described: nonmuscle invasive tumors and muscle-invasive bladder carcinomas, the latter being characterized by easy access to lymphatics and blood vessels for metastatic dissemination. In addition, low-grade or high-grade tumors can be described, characterized by different biological and clinical behaviors.

In the last years, major advances have been made in understanding the genetic mechanisms underlying BC. *FGFR3* or *p53* mutations seems to be mutually exclusive in urothelial carcinoma pathogenesis. In particular, *FGFR3* gene mutations selectively occur in noninvasive (pTa) BC, while *p53* mutations are rare (less than 5%) and are associated with high-grade tumors and invasive (pT1 or more) BC [72]. Generally, papillary pTa urothelial carcinomas show genetic stability, due to the absence of p53 inactivation, with chromosomal changes concerning only chromosome 9.

STAT3 has been implicated in the progression from carcinoma *in situ* to invasive BC. In particular, STAT3 signaling acts as an important downstream mediator of inflammatory cytokines, such as IL-6 and IL-17, which are released during bladder tumorigenesis due to chronic inflammation (i.e., smoking, persistent urinary tract infections) [73]. Moreover, early expansion of primitive CK14^+^ expressing cells, driven by STAT3 and other pathways, leads to transition to carcinoma *in situ*-invasive pathway [74] (Figure 1).

Zhang *et al*. investigated the inhibitory effects of STAT3 silencing on human T24 BC cells in *in vitro* and *in vivo* models. In this study, the downregulation of STAT3 or Survivin, an inhibitor of apoptosis, suppressed the proliferation of BC cells. Moreover, no additive effects were recorded by the STAT3 and Survivin joined knockdown, suggesting that they both belong to the same pathway in T24 cells [75].

CDC91L1, also called phosphatidylinositol glycan class U, is an oncogene overexpressed in BC and is an independent predictor of recurrence for nonmuscle invasive tumors. Cell division cycle 91-like 1 is activated by a chromosomal translocation and leads to persistent STAT3 activation [76].

EGF and its receptor EGFR are overexpressed in BC. In particular, EGFR expression is limited to the basal cell layer in the healthy urothelium, while it is expressed in both deep and superficial cell layers in both low- and high-grade BCs [77]. In response to EGF, STAT3 becomes activated and induces MMP-1 transcription by interacting with c-JUN, a component of the transcription factor activator protein-1 [78]. Interestingly, MMP-1 expression correlates with tumor high grade and invasiveness, thus suggesting that STAT3 activation is directly related to malignant behavior of T24 BC cells [79].

The establishment of a proangiogenic tumor environment is the consequence of a deregulated high expression of proangiogenic factors or an inadequate inhibition of angiostatic factors. Among the receptors for CXC chemokines, CXCR4, a receptor for CXC chemokine CXCL12/SDF-1, is involved in the maintenance of leukocyte trafficking during homeostasis and is key regulator of cell motility in BC. In the study led by Shen *et al*., higher CXCR4 expression correlated with STAT3 phosphorylation. In addition, STAT3 inhibitor Stattic inhibited CXCL12-triggered STAT3 phosphorylation and, consequently, cell motility and invasion in T24 BC cells [79].

## Role of STAT3 signaling in renal cell carcinoma

Renal cell carcinoma (RCC) is one of the most lethal urologic cancers [80]. In the last decade, a better understanding of the role of VEGF and mammalian target of rapamycin pathways has led to the introduction of several agents to the therapeutic landscape of metastatic RCC. Nevertheless, the rate of complete responses is still low [81], and alternative targets should be investigated in *in vitro* and *in vivo* studies.

STAT3 is involved in RCC carcinogenesis, growth and tumor angiogenesis (Figure 1). Masuda *et al*. quantified STAT3 and p53 mRNA expressions in a series of 47 Japanese patients with RCC. They found that the levels of STAT3 and p53 mRNA expressions were lower in tumor tissues compared with nontumor tissues and did not correlate with RCC histology and stage [82]. On the other hand, Guo *et al*. investigated the immunoprofile of phosphorylated STAT3 in a series of 88 RCCs with different histologies and 21 normal renal tissues. They reported that activated STAT3 was recorded in about 60% of ccRCC, 57% of papillary RCC and 33% of chromophobe RCC. Moreover, the nuclear expression of its phosphorylated form was enhanced in ccRCC, papillary RCC and urothelial carcinoma compared with tumor with inferior malignant potential (chromophobe) or benign tumor (oncocytoma) [83].

Furthermore, Zhou *et al*. analyzed the gene expression profiles of 60 clear cell RCC (ccRCC). Their results were matched with normal renal samples from The Cancer Genome Atlas. They found that fatty acid-binding protein 7 was one of the most commonly overexpressed genes in ccRCC and was able to promote tumor growth through the activation of STAT3 and ERK signaling pathways [84].

CD44 transmembrane glycoproteins is a ubiquitous cell surface adhesion molecule involved in cell–cell and cell–matrix interactions. CD44 was originally described as a mediator of lymphocyte homing to peripheral lymphoid tissues and is closely correlated with tumor cell proliferation, metastasis and patient outcome [85]. Qin *et al*. analyzed the expression of CD44 and activated STAT3 in a series of 75 RCC carcinoma and paired adjacent nontumor renal tissue samples from patients with localized ccRCC who underwent a nephrectomy. They found that CD44 was highly expressed in almost 50% or RCC and was associated with tumor grade, size and stage. They also observed a strong correlation between the expression of CD44 and pSTAT3. The simultaneous presence CD44 and pSTAT3 overexpression was recorded in 42.66% of tumor samples and had an additive negative impact on patient OS [86].

STAT3 has been shown to act as a potential modulator of HIF-mediated VEGF expression in RCC [87]. Activated STAT3 can increase HIF1α protein levels by blocking HIF1α degradation and enhancing its *de novo* synthesis. In addition, STAT3 can activate HIF1 target genes by binding to HIF1 target gene promoters and forming complexes with coactivators CREB-binding protein and p300, and RNA polymerase II [88]. In accordance with these findings, STAT3 inhibitor WP1066 has been shown to induce apoptosis, and inhibit the basal and hypoxia-induced expression of HIF1α and HIF2α, as well as VEGF secretion in RCC cell lines [89].

Tumor-associated macrophages play a vital role in the carcinogenesis and progression of RCC [90]. Tumor-associated macrophages release angiogenic cytokines that modulate RCC angiogenesis by activating NF-κB and STAT3 signaling. STAT3 is also involved in IL-6-induced proliferation of RCC cells [89] and in the activation of RCC cancer cells mediated by macrophages, thus representing an emerging target for the treatment of RCC.

STAT3 signaling pathway has been also associated with response to treatment in RCC. Single nucleotide polymorphisms in the STAT3 gene have been associated with better response to IFN-α [91]. On the other hand, inhibited STAT3 activity enhanced the antitumor effects of VEGFR-tyrosine kinase inhibitor sunitinib [92]. In addition, VEGFR-tyrosine kinase inhibitor axitinib has been shown to modulate antitumor immunity by downregulating STAT3 expression and reversing the RCC-induced immunosuppression mediated by myeloid-derived suppressor cells [93].

Recently, data concerning the inhibition of RCC growth and metastasis via AKT/mammalian target of rapamycin, ERK and JAK2/STAT3 pathway inhibition induced by simvastatin have opened a novel therapeutic perspective for these patients [94], also these data should be confirmed in randomized trials.

## Conclusion & future perspectives

Constitutively phosphorylated STAT3 has been implicated in several human tumors, including genitourinary cancers (Figure 1). Based on its involvement in tumor growth, metastasis and response to therapy, as well as in epigenetic regulation, CSCs and premetastatic niches, STAT3 has emerged as ideal molecular target for cancer therapy. The prominent role of STAT3, compared with other STAT members, as a therapeutic target for genitourinary cancer is sustained by its activity in both tumor, stromal and immune cells, which also promote tumor development and progression. In addition, the STAT3 inhibitors are associated with a potentially lower risk of related toxicities due to the transient status of STAT3 signaling in normal cells and to the minimal effects of STAT3 inhibition in mature cells.

STAT3 is placed at the crossroads of multiple signaling pathways. Small-molecule drugs to target STAT3 signaling have been developed over the last years and are in various phases of ongoing clinical study. Several strategies have been evaluated to target directly STAT3 or inhibiting STAT3 protein or indirectly through blockade of the upstream components of this pathway. However, a specific anti-STAT3 inhibitor is not yet clinically available. Furthermore, the combination of STAT3 inhibitors with approved or emerging antiangiogenic or immunotherapeutic agents should be investigated in patients with genitourinary tumors.

Extensive clinical studies are trying to identify anti-STAT3 drugs with high single-agent activity. However, the variety of STAT3 functions, together with its striking overall similarity with STAT1 and to the wide range of upstream activators converging on the JAK–STAT3 pathway, are enabling to identify a single target that may be effective for cancer therapy in patients with genitourinary tumors.

In conclusion, these data suggest that STAT3 signaling pathway is crucial in the carcinogenesis, growth, metastasis and response to treatment of genitourinary tumors. The identification and development of novel more selective agents will be an important scientific and clinical challenge in the therapeutic scenario of this population in the near future.

**Executive summary**

STAT3 promotes tumor growth, invasion and angiogenesis in prostate, bladder and renal tumors.
In prostate cancer, STAT3 activation correlates with pathological stage and Gleason score and modulates androgen receptor expression and function, as well as cancer stem cell and epithelial–mesenchymal transition.
Is involved in the resistance to enzalutamide.
Promotes the progression from carcinoma *in situ* to invasive bladder tumor.
In bladder cancer, STAT3 interacts with the EGF pathway and is associated with the expression of CD44, a glycoprotein involved in cell–cell and cell–matrix interactions.
In renal cell carcinoma, STAT3 cooperates with HIF1 to induce *VEGF* expression. STAT3 can increase HIF1α protein levels by blocking HIF1α degradation and enhancing its *de novo* synthesis.
